# Extracellular GAPDH Promotes Alzheimer Disease Progression by Enhancing Amyloid-β Aggregation and Cytotoxicity

**DOI:** 10.14336/AD.2020.1230

**Published:** 2021-08-01

**Authors:** Vladimir F Lazarev, Magda Tsolaki, Elena R Mikhaylova, Konstantin A Benken, Maxim A Shevtsov, Alina D Nikotina, Mirna Lechpammer, Vladimir A Mitkevich, Alexander A Makarov, Alexey A Moskalev, Sergey A Kozin, Boris A Margulis, Irina V Guzhova, Evgeny Nudler

**Affiliations:** ^1^Institute of Cytology of the Russian Academy of Sciences (RAS), Petersburg, Russia.; ^2^Engelhardt Institute of Molecular Biology, Russian Academy of Sciences, Moscow, Russia.; ^3^1^st^ University Department of Neurology, AHEPA hospital Aristotle University of Thessaloniki and Greek Alzheimer Association, Thessaloniki, Greece.; ^4^Saint Petersburg State University, Russia.; ^5^Klinikum rechts der Isar, Technische Universität München, Munich, Germany.; ^6^Department of Biochemistry and Molecular Pharmacology, New York University School of Medicine, New York, NY, USA.; ^7^Institute of Biology of Komi Scientific Centre of The Ural Branch of The Russian Academy of Sciences, Kommunisticheskaya, Russia.; ^8^Howard Hughes Medical Institute, New York University School of Medicine, New York, NY, USA.

**Keywords:** Alzheimer’s disease (AD), Glyceraldehyde-3-phosphate dehydrogenase (GAPDH), amyloid-β (Aβ)

## Abstract

Neuronal cell death at late stages of Alzheimer’s disease (AD) causes the release of cytosolic proteins. One of the most abundant such proteins, glyceraldehyde-3-phosphate dehydrogenase (GAPDH), forms stable aggregates with extracellular amyloid-β (Aβ). We detect these aggregates in cerebrospinal fluid (CSF) from AD patients at levels directly proportional to the progressive stages of AD. We found that GAPDH forms a covalent bond with Q15 of Aβ that is mediated by transglutaminase (tTG). The Q15A substitution weakens the interaction between Aβ and GAPDH and reduces Aβ-GAPDH cytotoxicity. Lentivirus-driven GAPDH overexpression in two AD animal models increased the level of apoptosis of hippocampal cells, neural degeneration, and cognitive dysfunction. In contrast, *in vivo* knockdown of GAPDH reversed these pathogenic abnormalities suggesting a pivotal role of GAPDH in Aβ-stimulated neurodegeneration. CSF from animals with enhanced GAPDH expression demonstrates increased cytotoxicity *in vitro*. Furthermore, RX-624, a specific GAPDH small molecular ligand reduced accumulation of Aβ aggregates and reversed memory deficit in AD transgenic mice. These findings argue that extracellular GAPDH compromises Aβ clearance and accelerates neurodegeneration, and, thus, is a promising pharmacological target for AD.

For decades, Alzheimer disease (AD) has been defined as a dementia syndrome confirmed by postmortem neuropathological observation of neuritic plaques and neurofibrillary tangles, which are known to be composed, respectively, of amyloid-β (Aβ) and paired helical filament tau [1]. Recent large genome-wide association meta-analysis of 94,437 individuals with clinically diagnosed AD, has shown that genetic variants with affected processing of Aβ and amyloid precursor protein are associated with both early-onset autosomal dominant AD and with late-onset AD [2]. The amyloid hypothesis of AD suggests that the imbalance between production and clearance of Aβ in the human brain is critical in AD pathogenesis [3]. Accordingly, AD pathogenesis is triggered by the formation of soluble oligomers from physiologically monomeric Aβ molecules, followed by the generation of insoluble polymeric Aβ aggregates, eventually accumulating as amyloid plaques [4]. Aβ oligomers are known to injure synapses, which leads to microglial and astrocytic activation, causing an inflammatory response and disruption of neuronal ionic homeostasis; the latter leading to oxidative injury and altered kinase/phosphatase activities that cause the formation of tangles [5,6]. The outcome of this pathologic chain of events is synaptic dysfunction and massive neuronal loss. Given the clinical hallmarks of AD - formation of polymeric Aβ aggregates and accumulation of amyloid plaques - novel, investigative therapeutic approaches are currently focusing on the design of peptides capable of binding to Aβ to block either fibril formation or Aβ elongation in order to prevent the formation of monomers/oligomers [7].

Neuronal death during AD causes the release of cytosolic proteins, some of which may interact with endogenous Aβ. It has been hypothesized that the glycolytic enzyme glyceraldehyde-3-phosphate dehydrogenase (GAPDH), one of the most abundant cellular proteins, may have a role in AD pathogenesis [8,9], possibly due to the formation of neurotoxic aggregates with Aβ [10,11]. Tissue transglutaminase (tTG) has been also linked to AD pathology [12]. This enzyme catalyzes the formation of intermolecular covalent bonds between surface lysines of GAPDH and glutamine residues of partner proteins, such as mutant huntingtin [13,14]. GAPDH was also found to increase cytotoxicity and prion-like properties of pathogenic poly-Q proteins [15,16].

**Figure 1.**
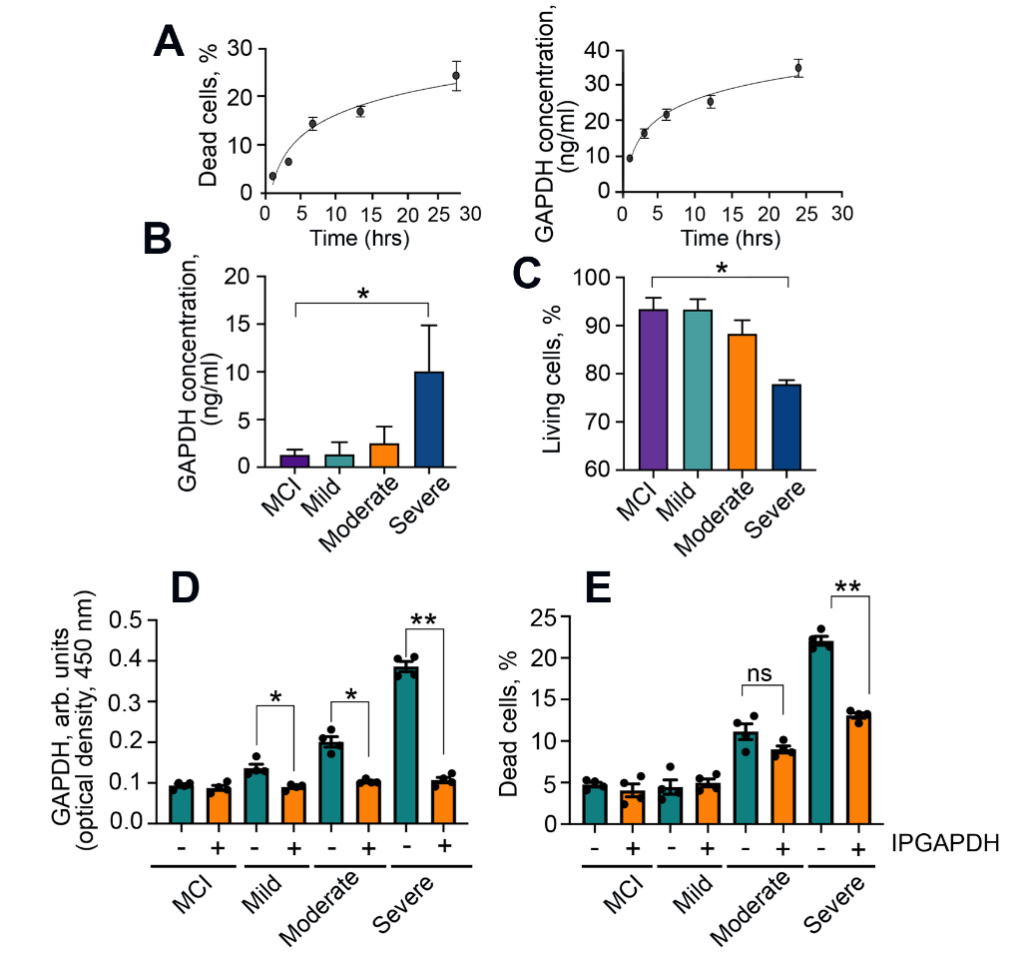
The level of GAPDH correlates with the stage of AD. (A) The level of SH-SY5Y dead cells incubated with Aβ42 (6 μM) for the indicated times (left panel). Levels of GAPDH measured in the same cell growth media with a two-site (sandwich-type) ELISA (right panel). (B) Levels of GAPDH measured in CSF of patients with MCI, mild, moderate, and severe stages of AD; * p < 0.01. (C) SH-SY5Y cells were incubated with CSF from (*B*) for 48 h, and the amount of cell death was estimated using the CytoTox-96 assay; *p < *0.01*. (D, E) CSF samples of AD patients were depleted of GAPDH using 6C5 antibody, as detected by two-site ELISA (D); their cytotoxicity determined with the Cytotoxic assay (E), *p < 0.05, **p <0,01.

As Aβ contains glutamine residue at position 15 (Q15) that could potentially be targeted by tTG [17], we examined whether the aggregation of Aβ with GAPDH in the presence of tTG resulted in the covalent Aβ-GAPDH complex formation. We found a direct correlation between the amount of such complexes and the stage of the disease and established the cytotoxic nature of Aβ-GAPDH *in vitro*. Furthermore, we found that the increased level of constitutive GAPDH in AD animal models results in aggravation of cognitive deficits and brain pathology linked to AD progression, which can be rescued by a specific GAPDH-targeting small ligand. These findings establish GAPDH as a promising AD therapeutic target.

## MATERIALS AND METHODS

### Patients and CSF collection

CSF samples were collected via spinal tap from 190 patients (107 male, 83 females; average age 75,3 ± 0,5) between 2007 and 2017 at the Care Units for Alzheimer’s Disease Problems ‘Saint Eleni’ and ‘Saint Ioannis’ in Thessaloniki, Greece. The diagnosis was established according to the NINCDS-ARDRA criteria for AD [18], and the Petersen criteria for MCI [19]. Patients were assigned to severity subgroups based on their scores on the Mini-Mental State Examination (MMSE) [20]. CSF samples were collected via spinal tap in 10 mL polypropylene tubes and stored at -80 °C within 1 h after sampling. CSF samples from the first patient cohort, used for GAPDH detection, were centrifuged at 2000 *g* for 10 min at 4 °C before freezing (Fig. 1F). CSF samples from the second patient cohort, used for detecting Aβ-GAPDH aggregates, were frozen without centrifugation (Fig. 1G). Samples were delivered to the Institute of Cytology of Russian Academy of Sciences, St. Petersburg, Russia, in June 2016 and October 2017, respectively, on dry ice for further analyses. All CSF samples had only one freeze-thaw cycle.

The first cohort comprised of four patient groups: (1) mild cognitive impairment (MCI) *n* = 22; (2) mild AD-type dementia (‘Mild’), *n* = 41; (3) moderate AD-type dementia (‘Moderate’) *n* = 49; and (4) severe AD-type dementia (‘Severe’), *n* = 51. The second cohort represented the same patient groups: (1) n = 6; (2) n = 8; (3) n = 7; (4) n = 6.

For all patients participating in the study a written informed consent was obtained either directly from the capable individuals or from legally authorized representatives, according to the Declaration of Helsinki [21], and the Research Ethics Committee approvals at each center participating in the study.

### CSF analysis for GAPDH

To measure the amount of GAPDH in conditioned medium and in CSF samples, a previously-developed double-site sandwich-like ELISA immunoassay was used [16]. Calibration standards of pure GAPDH and CSF samples diluted 1:9 in PBS were applied to the wells for 3 h, after which they were washed. Monoclonal 6C5 anti-GAPDH was then added, followed by secondary anti-mouse horseradish peroxidase-conjugated antibody (Jackson Immuno-Research, USA).

### CSF analysis for Aβ-GAPDH aggregates

Previously frozen, non-centrifuged CSF samples were analyzed using the filter-trap assay [22]. For each sample, 250 µL of CSF was mixed with 250 μL of buffer containing 20 mM Tris HCl, pH 7.5, 20 mM NaCl, and 4% sodium dodecyl sulfate (SDS). The mixtures were sonicated and ultra-filtered with the use of a dot-blotting manifold (BioRad, USA). The membranes were incubated with antibodies against either Aβ (Abcam, UK) or GAPDH (Abcam, UK), and subsequently with secondary antibodies conjugated with peroxidase; visualization of the dots was performed using a chemiluminescence protocol. Dot intensity was measured with TotalLab Quant software.

Fönster resonance energy transfer (FRET) was used to analyze the amount of Aβ-GAPDH in CSF samples. Briefly, 100 µL of CSF samples were diluted in 100 µL of PBS, then antibodies were added. Rabbit anti-Aβ antibody (Sigma-Aldrich, USA) was labeled with Alexa488 fluorescent dye (Thermo Fisher Scientific, USA); mouse anti-GAPDH antibody (Clone 6C5, Abcam, UK) was conjugated with CF555 fluorescent dye (Sigma-Aldrich, USA). All antibodies were diluted in the ratio 1:200. The mixtures containing CSF sample, PBS and indicated antibodies were incubated for 1 h, then the fluorescence emission spectrum of each sample was determined using a Varioscan LUX device (Thermo Fisher Scientific, USA). The fluorescence emission spectrum was detected from 515 nm to 600 nm at the excitation wavelength of 490?nm. Then fluorescence emission at 568 nm at the excitation wavelength of 490?nm was separately measured. For all data, the final fluorescent signals were obtained by subtracting the fluorescent signals with the background noise from a blank well (200 µL of PBS containing all antibody set without CSF samples). The experiments were repeated for four times and the average value of fluorescence were taken at each specific condition.

To analyze the toxicity of CSF samples, 50 μL of CSF was mixed with 50 μL of cell culture medium, added to SH-SY5Y cells in a 96-well plate and incubated for 48 h. Cell viability was measured using the 3-(4,5-dimethylthiazol-2-yl)-2,5-dimethyltetrazolium bromide (MTT) assay as previously described [23].

### Cells, plasmids, peptides, and proteins

Human neuroblastoma SY-SH5Y cells (ATCC, USA) were grown in Dulbecco's modified Eagle's medium, supplemented with the nutrient mixture F-12, L-glutamine (Gibco, USA), 10% fetal calf serum (PAA Laboratories GE, Austria), and 50 mg/mL of gentamicin (Biolot, Russia) under 5% CO_2_ at 37 °C. Cell death was estimated using the CytoTox-96 assay (Promega, USA) based on measurements of lactate dehydrogenase (LDH) activity in the culture medium.

To vary the GAPDH level in brain cells, plasmids bearing the entire *gapdh* gene, pLOC-GAPDH (GAPDH knock-in), and clone TRCN0000041460, shRNA to GAPDH (GAPDH knock-down), mature antisence: CTGAGTATGTCGTGGAGTCTA were purchased from Termofisher Scientific (USA). Packaging (Δ8.91) and envelope (pVSV-G) plasmids were purchased from Invitrogen, (USA). The HEK-293T host cells were transfected using polyethyleneimine (Sigma, USA) mixed with all three plasmids.

Aβ42, Aβ16, Aβ42^Q15A^, Aβ16^Q15A^, and biotinylated Aβ42 were purchased from GeneCust (Switzerland). GAPDH isolated from rabbit muscle was kindly provided by Dr. Muronetz (Moscow State University, Moscow, Russia).

### Protein-protein interaction assays

All tests for the interaction (i.e., co-aggregation) of GAPDH and Aβ peptides were performed under the following conditions: 30 μg/mL Aβ peptide, 30 μg/mL GAPDH, and 1 μg/mL tTG, dissolved in buffer containing 20 mM Tris HCl, pH 7.5, 20 mM NaCl, and 10 mM CaCl_2_. Incubations were at 37°C for 18 h for electrophoresis or 48 h for the filter-trap assay and atomic force microscopy (AFM).

To analyze the interaction between GAPDH and biotinylated Aβ42, we developed a new ELISA-based test. Purified GAPDH was diluted to a concentration of 4 mg/mL in PBS and immobilized for 1 h on an F96 MicroWell™ plate (Nunc, Denmark). The plate was washed with PBS and blocked for 1 h with PBS containing 3% fetal calf serum. Immobilized GAPDH was then incubated for 1.5 h with 30 μg/mL Aβ42 dissolved in a buffer containing 20 mM Tris HCl, pH 7.5, 20 mM NaCl, and 10 mM CaCl_2_. Factors affecting the interaction between GAPDH and Aβ42, such as transglutaminase (tTG) or cystamine, were added at this stage. For detection, avidin-peroxidase conjugate (Sigma-Aldrich, USA) was used. Visualization was performed using the tetramethylbenzidine protocol. The estimation of the interaction between GAPDH and non-biotinylated Aβ peptides was carried out using competitive inhibition of biotinylated Aβ42 binding to immobilized GAPDH. All experiments were performed in triplicate.

### Atomic force microscopy

Sample protein mixtures prepared as described above were placed on glass supports, dried, and analyzed with the aid of a Ntegra Aura microscope (NT-MDT, Russia). The atomic-force studies were conducted at the Resource Centre for Microscopy and Microanalysis, St. Petersburg State University.

### Animals

Two animal models of AD were used in this study: (1) “chemical” model in Wistar rats and (2) transgenic 5XFAD mice.

### Chemically-induced model of Alzheimer's disease in rats

The chemically-induced model of AD in rats [24] is widely used in AD studies. Briefly, Wistar male rats at P90-P100 weighing 200-220 g were divided into 7 groups (*n* = 9 in each group) as follows: untreated, sham-operated, injected with Aβ42, injected with shGAPDH RNA lentiviral construct, injected with pLOC-GAPDH lentiviral construct, injected with Aβ42 + shGAPDH RNA lentiviral construct, and injected with Aβ42 + pLOC-GAPDH lentiviral construct. Aβ42 and the viral constructs were dissolved in 5 mL of sterile, phosphate-buffered saline (PBS). Injections were carried out using a stereotaxic device (SR-5R, Narishige Scientific Instrument Laboratory, Japan) at the following coordinates: anteroposterior (AP) = -4 mm, lateral (L) = 2.5 mm, and dorsoventral (DV) = 3 mm [25,26]. Before the surgery rats were anesthetized with 10 mg “Zoletyl-100” (tiletamine hydrochloride and zolazepam, Carros Cedex, France) and 0.2 ml 2% Rometar (xylazinum hydrochloride, Bioveta, Czech Republic) injected intraperitoneally before being mounted in a stereotactic frame. We anesthetized the mice with 1 mg "Zoletyl-100" and 0.02 ml 2% Rometar in 0.1 ml normal saline. All *in vivo* experiments were carried out following the requirements of the Institute of Cytology of Russian Academy of Sciences ethic committee (Identification number F18-00380).

### Transgenic model of AD

The 5XFAD transgenic mice were genotyped by PCR analysis of DNA extracted from ear biopsies. The transgenic cassette was detected using primers 5’-AGGACTGACCACTCGACCAG-3’ and 5’-CGGGGGTCTAGTTCTGCAT-3’, yielding a 377 bp product. Siblings of 5XFAD mice were used as wild-type (wt); this protocol was described by Peters et al [27].

Wt and 5XFAD male mice at P60 were divided into 8 groups of 6 animals as follows: 1) untreated wt, 2) sham-operated wt, 3) wt injected with the shGAPDH RNA lentiviral construct, 4) wt injected with the pLOC-GAPDH lentiviral construct, 5) untreated 5XFAD, 6) sham-operated 5XFAD, 7) 5XFAD injected with the shGAPDH RNA lentiviral construct, and 8) 5XFAD injected with the pLOC-GAPDH lentiviral construct. Viral constructs were dissolved in 5 μL of sterile PBS and injected using a stereotaxic device with a special adapter (SR-5R, Narishige Scientific Instrument Laboratory, Japan) at the following coordinates: AP = -2.5 mm, L = 1.5 mm, and DV = 1.7 mm. Coordinates were determined accordingly to The Mouse Brain Library service (mbl.org) [28].

### Morris water maze test

Memory impairment in rats (n=9) and mice (n=6) from each experimental group was evaluated using a Morris water-maze test [29] of diameter 1.5 m (OpenScience, Russia) on day 60 after Aβ42 injection into rats and on P180 for 5XFAD mice. The time required for experimental animals to find the submerged platform was measured. The swimming trajectories of animals in experiments with the hydrocortisone treatments were treated according to EthoVision XT14.0. [30].

### Magnetic resonance imaging (MRI)

The brains of 3 randomly selected rats from each experimental group were analyzed by MRI. The assessment of the parenchymal brain damage induced by the stereotactic injections of Aβ peptides and lentiviruses employed a high-field (9.4 T) magnetic-resonance scanner (Avance III 400 WB; Bruker, Germany) with the following protocols: FLASH, Rare T1, and TurboRare T2. All images were obtained with a slice thickness of 1.0 mm in the coronal plane. Assessment of the MR scans was assisted by the Paravision 5.1 software package (Bruker BioSpin GmbH, Rheinstetten, Germany) and Analyze software (AnalyzeDirect, Inc., Overland Park, KS, USA) [22].

### Analysis of apoptosis

Brains of 3 randomly selected rats and / or mice from each experimental group, as described above were used for immunohistological analysis. Histological preparations of frontal sections at the hippocampus level (12 μm) were stained with the Click-IT TUNEL kit (Life Technologies, USA).

### Statistical analysis

All numeral results are reported as the mean ± standard error of the mean (SEM) and represent data from a minimum of three independent experiments. Statistical analysis was performed using the Nonparametric Mann-Whitney U-test for groups that did not pass the Normality test (Shapiro-Wilk method). These data were processed using Statistica Version 13.3 for Windows. For data that had a Gaussian distribution, the one-way ANOVA test followed by Tukey’s comparison test (when compare the mean of each condition with every other comparison) was used with the aid of Graph Pad Prism 8.0 (Graph Pad Software Inc). Differences were considered statistically significant at *p* < 0.05.

## RESULTS

### GAPDH in CSF contributes to Aβ toxicity

To test whether GAPDH is released from cells affected by exogenous Aβ, we utilized a human neuroblastoma cell line, SH-SY5Y [31]. The cells were incubated with Aβ42 peptide for indicated times, and the profiles of cell death, as determined by lactate dehydrogenase activity in culture medium, and the extracellular GAPDH concentration, as measured using a sandwich-type ELISA, were compared (Fig. 1A). The matching profiles indicate that the release of GAPDH in the intercellular space is coupled to the toxic effect of exogenous Aβ42.

To determine whether GAPDH progressively accumulates in CSF of AD patients, we studied samples from first cohort of patients with Mild Cognitive Impairment (MCI) (n = 22), as well as with mild (n = 41), moderate (n = 49), and severe (n = 51) AD, stratified based on Mini-Mental State Examination (MMSE) scores [20] (Supplementary Table 1). The level of GAPDH measured with the sandwich-type ELISA immunoassay was low in samples from MCI and ‘mild AD’ patients, and progressively increased with the AD stage, reaching 12.5 ± 1.5 ng/mL in the ‘severe AD’ group (Fig. 1B).

To determine whether the extracellular GAPDH impacted cell death, 50 μL of each CSF sample was mixed with 50 μL of growth medium and added to SH-SY5Y cell cultures. Cell death increased progressively as a function of AD stage, reaching 19.2 ± 0.9% for samples from the ‘severe AD’ group (Fig. 1C). Depleting CSFs of GAPDH using specific antibody (Fig. 1D) diminished CSF-induced cell death (Fig. 1E), indicating that GAPDH contributes to CSF toxicity.

**Figure 2.**
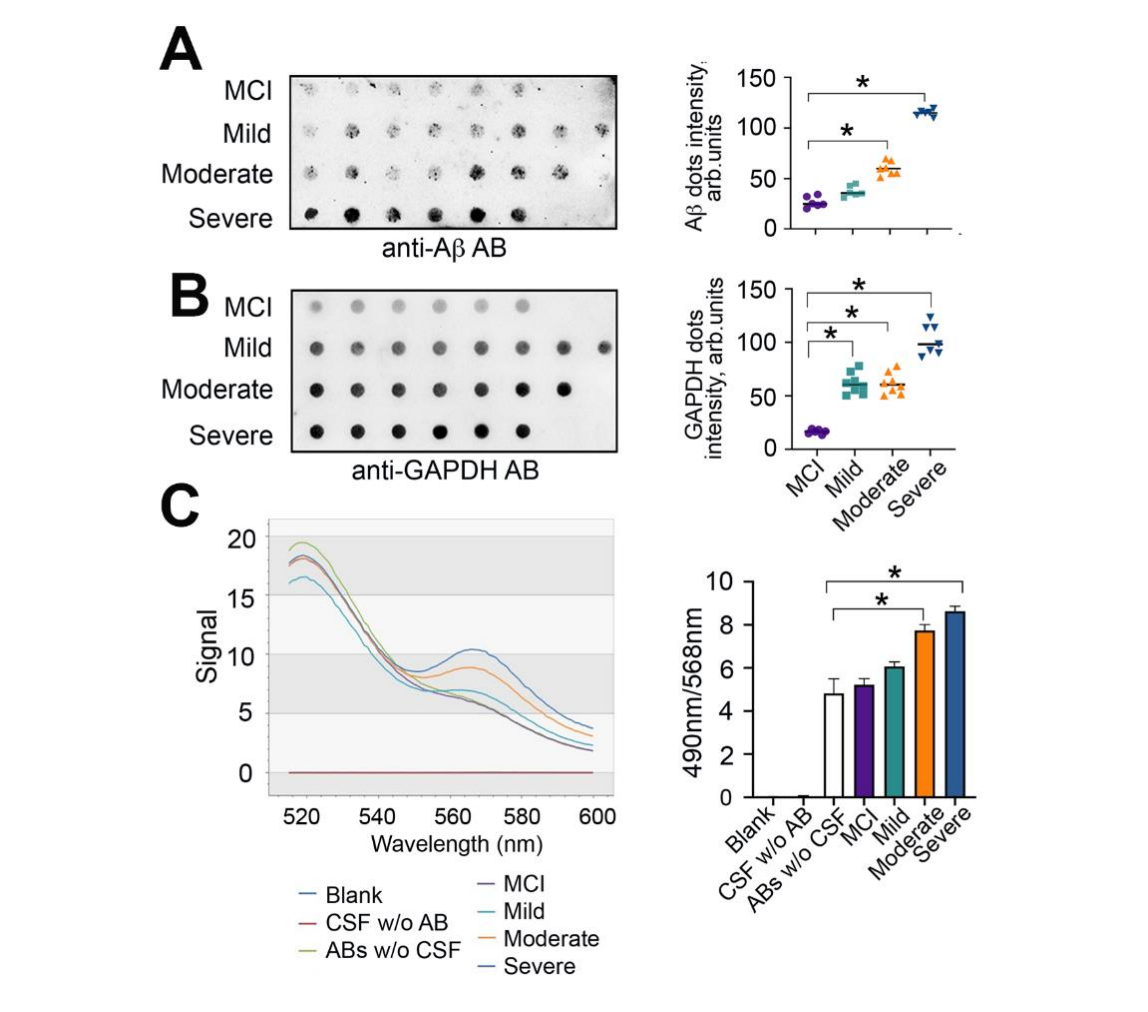
Aβ-GAPDH complex formation as a function of AD progression. (A) and (B) CSF analysis by the filter-trap assay. The representative filter was probed with antibodies against Aβ (A, left panel) or GAPDH (B, left panel). The quantitation of blots using TotalLab software (right panels); * *p < 0.01*. (C) FRET analysis of GAPDH-Aβ interaction in CSF samples from AD patients. Representative emission spectra detected at 516-600 nm, excitation 488 nm (left panel) and quantitation of the FRET signals at excitation/emission 490/568 nm (right panel); * *p < 0.01*.

We next investigated the aggregation status of GAPDH and Aβ42 in CSF. Samples from the second cohort from MCI (*n* = 6), mild AD (*n* = 8), moderate AD (*n* = 7), and severe AD (*n* = 6) groups, were examined (Supplementary Table 2). To prevent the loss of aggregates we avoided centrifugation. Instead, the sodium dodecyl sulfate (SDS)-insoluble aggregating material was collected by ultrafiltration using a 96-well dot-blotting manifold; the membrane was stained with an antibody against Aβ (Fig. 2A), and another identical 96-array dot-blot was stained with an anti-GAPDH antibody (Fig. 2B). The number of Aβ42-GAPDH co-aggregates trapped on the filter strongly correlated with the disease stage. The difference in dot density between MCI samples and samples from patients with severe AD was 4-fold for Aβ42 (Fig. 2A) and 7-fold for GAPDH (Fig. 2B).

To determine whether extracellular GAPDH forms a complex with Aβ in CSF of AD patients we used Fönster resonant energy transfer (FRET). To detect photon transfer from a fluorophore-donor, Alexa 488 conjugated with Aβ antibody, to a fluorophore-acceptor, CF-555 conjugated with GAPDH antibody, we obtained an emission spectrum in the range of 516-600 nm at an excitation wavelength 490 nm. We found a progressive increase in FRET signal at 568 nm, which directly correlated with the disease stage (Fig. 2C). Although the change in the FRET signal was noticeable in CSF from ‘Mild AD’ patients (6,1 ± 0,2 vs 4,8 ± 0,7 arb.un), it was not statistically significant. However, FRET changes for samples from ‘Moderate’ and ‘Severe’ AD patients were significantly higher than in the control (Fig. 2C). We conclude that the extracellular GAPDH forms a complex with Aβ in CSF of AD patients and the more advanced the stage of the disease the higher is the concentration and toxicity of these complexes.

### Covalent bonding between Aβ42 and GAPDH promotes toxic co-aggregation

Our findings that Aβ42 and GAPDH persist as SDS-insoluble aggregates in the CSF of patients with AD and previous reports linking tTG to AD and Huntington disease pathology [12,32] prompted us to examine the possibility of tTG-mediated covalent interaction between the two polypeptides.

**Fig. 3.**
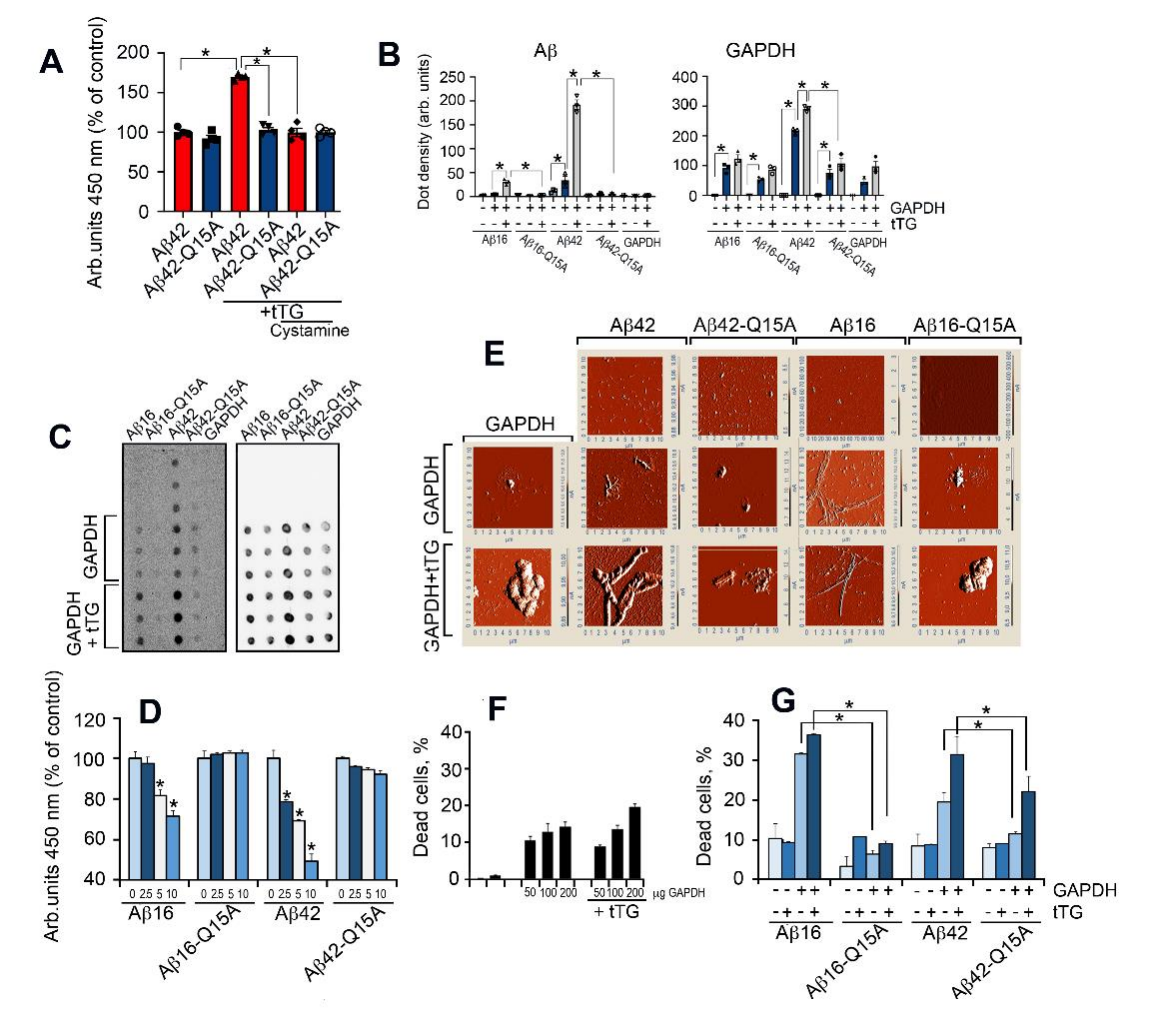
Covalently bound Aβ42 and GAPDH are highly cytotoxic. (A) Results of the GAPDH-Aβ42 interaction assay. GAPDH was immobilized at the bottom of a 96-well plate followed by the addition of biotinylated Aβ42 with tTG alone or together with its inhibitor cystamine; *, *p < 0.05*. (B) Filter-trap assay results for the complexes of Aβ16, Aβ16^Q15A^, Aβ42, or Aβ42^Q15A^ with GAPDH formed in 48 h. * *p <0.01*. (C) Representative dot density from (*B*); light blue bars indicate Aβ, dark blue - Aβ with GAPDH, gray - Aβ with GAPDH and tTG; (D) Results of the competitive protein-protein interaction assay. GAPDH was immobilized at the bottom of a 96-well plate, and biotinylated Aβ42 was added to the wells together with non-labeled Aβ42, Aβ16, Aβ42^Q15A^, and Aβ16^Q15A^ in the following concentrations: 0 μg (light blue bars), 2.5 μg (dark blue bars), 5 μg (white bars) and 10 μg (bright blue bars). (E) AFM of the protein mixtures prepared as in (B). (F) GAPDH-Aβ42 cytotoxicity. SH-SY5Y cells were incubated with tTG (5 μg/mL) or with GAPDH in various concentrations (left panel). (G) SH-SY5Y cells were incubated with complexes of GAPDH (30 μg/mL) with Aβ peptides (6 μM) in the presence or absence of tTG (5 μg/mL); white bars depict toxicity of Aβ alone, bright blue - with tTG, light blue - with GAPDH, and dark blue - with GAPDH and tTG. Cell death was estimated with the CytoTox-96 assay (right panel); * *p <0.05*.

tTG-mediated bond formation may occur between surface exposed lysines of GAPDH and glutamine 15 of Aβ42 [33]. To test this possibility, we designed a protein-protein interaction assay in which GAPDH was immobilized at the bottom of a 96-well plate, and then biotinylated Aβ42 or Aβ42 with a Q15A substitution (Aβ42^Q15A^) were added in the presence of tTG alone, or together with cystamine, a tTG inhibitor, followed by washing. tTG enhanced the binding of GAPDH to Aβ42, but not to Aβ42^Q15A^, by approximately 50%, whereas cystamine abolished this effect (Fig. 3A).

To further investigate the stable association between GAPDH and Aβ42, the mixtures containing GAPDH, Aβ42, tTG, and cystamine were prepared in combinations as shown in Supplementary Fig. 1, incubated for 12 hours, followed by SDS gel separation and Western blotting (Supplementary Fig. 1). The only visible band stained with anti-Aβ antibody has the same mobility as GAPDH (36 kDa) (upper panel). This band, which appeared after gel separation of the mixture containing GAPDH, Aβ42, and tTG, constitutes the SDS-insoluble complex of Aβ-GAPDH.

The N-terminal fragment (residues 1-16) appears to be the minimal part of Aβ capable of triggering amyloidosis *in vivo* [34]. We therefore examined the ability of Aβ16 and Aβ16^Q15A^, as well as Aβ42 and Aβ42^Q15A^, to bind GAPDH in the presence or absence of tTG using a filter-trap assay *(*Fig. 3B). Both Aβ16 and Aβ42 formed co-aggregates with GAPDH (Fig. 3 B, C). The level of aggregation was much higher in the presence of tTG. Q15A substitution greatly reduced the ability of Aβ42 and Aβ16 to form such aggregates (Fig. 3 B, C).

To further establish the role of Q15 in the Aβ-GAPDH complex, a competition protein-protein interaction assay was designed in which GAPDH was immobilized at the bottom of a 96-well plate, and biotinylated Aβ42 was added to the wells together with non-tagged Aβ peptides at various concentrations in the presence of tTG (Fig. 3D). Aβ42 and Aβ16 peptides, but not their Q15A derivatives, reduced the binding of biotinylated Aβ42 to GAPDH in a dose-dependent manner *(*Fig. 3D), thus directly supporting the role of Q15 in tTG-mediated Aβ-GAPDH complex formation.

We next employed atomic force microscopy (AFM) to examine the structure of GAPDH-Aβ complexes (Fig. 3E). Aβ42 forms only tiny fibril-like structures, whereas Aβ16 does not form any visible fibrils. However, in the presence of GAPDH, and especially in combination with tTG, the fibril-like structures become obvious; their size increases for both Aβ42 and Aβ16. Notably, the aggregates found in the GAPDH mixtures with Aβ16^Q15A^ or Aβ42^Q15A^ were amorphous, closely resembling those produced by GAPDH alone (Fig. 3E).

As shown in Fig.1C, CSF samples from AD patients are toxic to SH-SY5Y cells. To test whether Aβ peptides with a Q15A substitution retain their toxicity, cultures of SH-SY5Y cells were incubated with Aβ42/16 peptides, or their Q15A derivatives, along with GAPDH or GAPDH+tTG. GAPDH alone or with tTG was minimally toxic (Fig. 3 F, G). The tTG enzyme itself was not toxic. The number of dead cells significantly increased in the presence of Aβ42/16 and GAPDH (19.5 ± 2.4% and 31.6 ± 0.3%, respectively) and further increased in the presence of tTG (31.5 ± 4.6% and 36.1 ± 0.4% for Aβ16 and Aβ42, respectively). Q15A derivatives demonstrated much less toxicity under the same conditions (Fig. 3G).

Finally, we examined whether a protein other than GAPDH, which can also be released from dying neurons, formed insoluble complexes with Aβ42. Lactate dehydrogenase (LDH) is an abundant enzyme that is solely cytoplasmic and serves as a common indicator of cell death if found intracellularly [35]. We used utrafiltration to compare the abundance of aggregates formed by Aβ42 in the presence of GAPDH+tTG or LDH+tTG (Fig. S2). The amount of Aβ42 aggregates formed in the presence of LDH+tTG did not differ statistically from those formed by Aβ42 alone (adjusted P value 0.2365). In contrast, Aβ42 aggregates formed with GAPDH were at least four-fold more abundant than those obtained with LDH (Supplementary Fig. 2).

**Figure 4.**
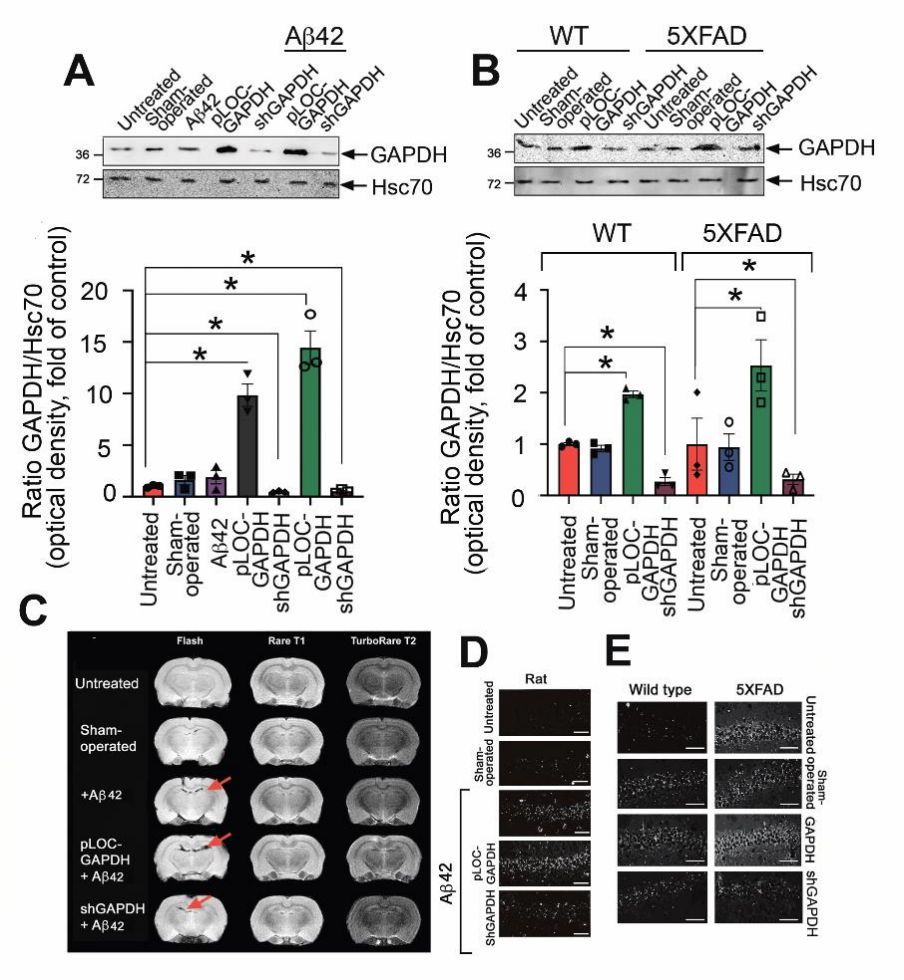
GAPDH in the hippocampus promotes brain injury in AD animal models. (A) Representative Western blots of hippocampal samples from rats injected with vehicle (*n* = 3, sham-operated), lentiviral pLOC-GAPDH plasmid (*n* = 3), or lentiviral shGAPDH RNA (*n* = 3), with or without Aβ42. Hsc70 antibody was used as loading control (upper panel). GAPDH/Hsc70 ratio is shown as a fold change to control (untreated) for three animals in each experimental group (lower panel). (B) GAPDH western blots of hippocampal samples from WT and 5XFAD mice injected with vehicle (*n* = 3, sham-operated), lentiviral pLOC-GAPDH plasmid (*n* = 3), or lentiviral shGAPDH RNA (*n* = 3) (upper panel); quantitation of relative band intensity (lower panel). (C) Representative magnetic resonance brain images (of rats two months after injection of Aβ42 together with lentiviral pLOC-GAPDH or Aβ42 together with lentiviral shGAPDH RNA). MRI protocols: Rare T1, TurboRare T2, and FLASH (gradient echo (D) Representative frontal histological slices of hippocampus from experimental animals stained with the Click-IT TUNEL kit (*n* = 3 in each experimental group); Scale bar = 100 μm. (D) Representative histological slices (upper panel) of hippocampi of wt and 5XFAD mice, sham-operated or injected with lentiviral pLOC-GAPDH or shGAPDH RNA; Scale bar = 100 μm.

**Figure 5.**
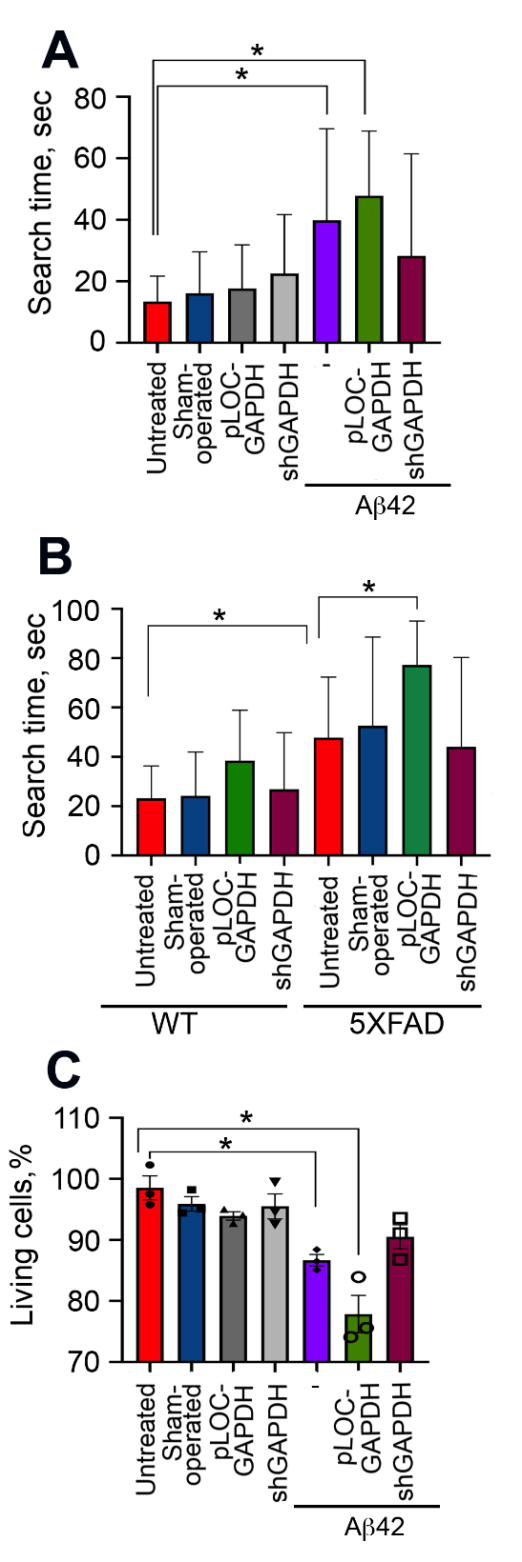
GAPDH in hippocampus of AD animals impairs spatial memory and increases CSF cytotoxicity. (A) Morris water-maze results for rats tested two months after surgery following the administration of lentiviral pLOC-GAPDH or shGAPDH RNA with or without Aβ42. (B) Morris water-maze results for 6-month-old wt and 5XFAD mice treated as in (A). (C) SH-SY5Y cells were incubated with CSF from rats in (*A*) for 24 h, and the level of cell death was estimated with the CytoTox-96 assay; * *p < 0.05*.

### Extracellular GAPDH in murine hippocampus exacerbates AD pathology

As shown above, the extracellular complex of GAPDH-Aβ is cytotoxic for cultured neuronal cells. To test whether this would also be the case in mammals *in vivo*, we adopted two animal AD models: a chemical model, in which exogenous Aβ42 was introduced into the rat hippocampus, and a genetic model using 5XFAD mice. Animals were surgically infected with lentiviruses bearing either a plasmid overexpressing GAPDH (pLOC-GAPDH) or shRNA targeting GAPDH to generate, respectively, animals with high or low GAPDH expression in the hippocampus. Nine weeks after lentivirus administration, GAPDH levels in the hippocampi of the animals were estimated by immunoblotting. Rats infected with shGAPDH or pLOC-GAPDH displayed, respectively, a 69% decrease or 9-fold increase in GAPDH, as compared to untreated animals (Fig. 4A). Similar down- and up-regulation of GAPDH occurred in the wild-type (wt) and 5XFAD mice (Fig. 4B).

MRI was used to estimate the damage to the rat hippocampus caused by Aβ42 introduced into the CA1 area. Image analysis showed that the injury zone in Aβ42-treated animals positively correlated with the GAPDH level in the hippocampus (Fig. 3C). The ratio between the lesion volume and the total hippocampus was the highest in the pLOC-GAPDH+Aβ42 group (14.98%) and minimal in the shGAPDH+Aβ42 group (2.88%), which is similar to that in sham-operated rats (2.52%) (Fig. 4 C and Supplementary Fig. 3A).

To determine whether the tissue damage was due to apoptotic death of hippocampal cells, histological sections of the hippocampus were stained with the TUNEL assay. The level of apoptosis positively correlated with the level of GAPDH (Fig.4 D and Supplementary Fig. 3B). The stereotactic surgery procedure caused some cell death in the brain; the number of apoptotic cells in operated animals in all control groups was approximately 20%. However, injection of Aβ42 caused the accumulation of 39.6 ± 3.3% apoptotic cells while the injection of pLOC-GAPDH+Aβ42 caused the accumulation of 45.6 ± 4.9% apoptotic cells (Fig.4 D and Supplementary Fig. 3B). Remarkably, the level of apoptosis in the brains of rats injected with shGAPDH+Aβ42 was similar to that of sham-operated animals (24.5 ± 0.3%) (Fig.4 D and Supplementary Fig. 3B).

Analogous to the rat model, the level of apoptotic cells in the hippocampus of 5XFAD mice was elevated 3.5-fold by the injection of pLOC-GAPDH, as compared to untreated 5XFAD mice (35.1 ± 0.5 vs. 10.5 ± 0.7) (Fig. 4E and Supplementary Fig. 3C). The number of apoptotic cells in mice with knocked-down GAPDH was close to that of sham-operated 5XFAD mice (13.2 ± 1.7% vs. 10.5 ± 0.7%) (Fig. 4E and Supplementary Fig. 3C). Thus, the level of GAPDH in the hippocampus of AD-simulating animals directly correlates with the level of apoptosis associated with AD pathology.

**Figure 6.**
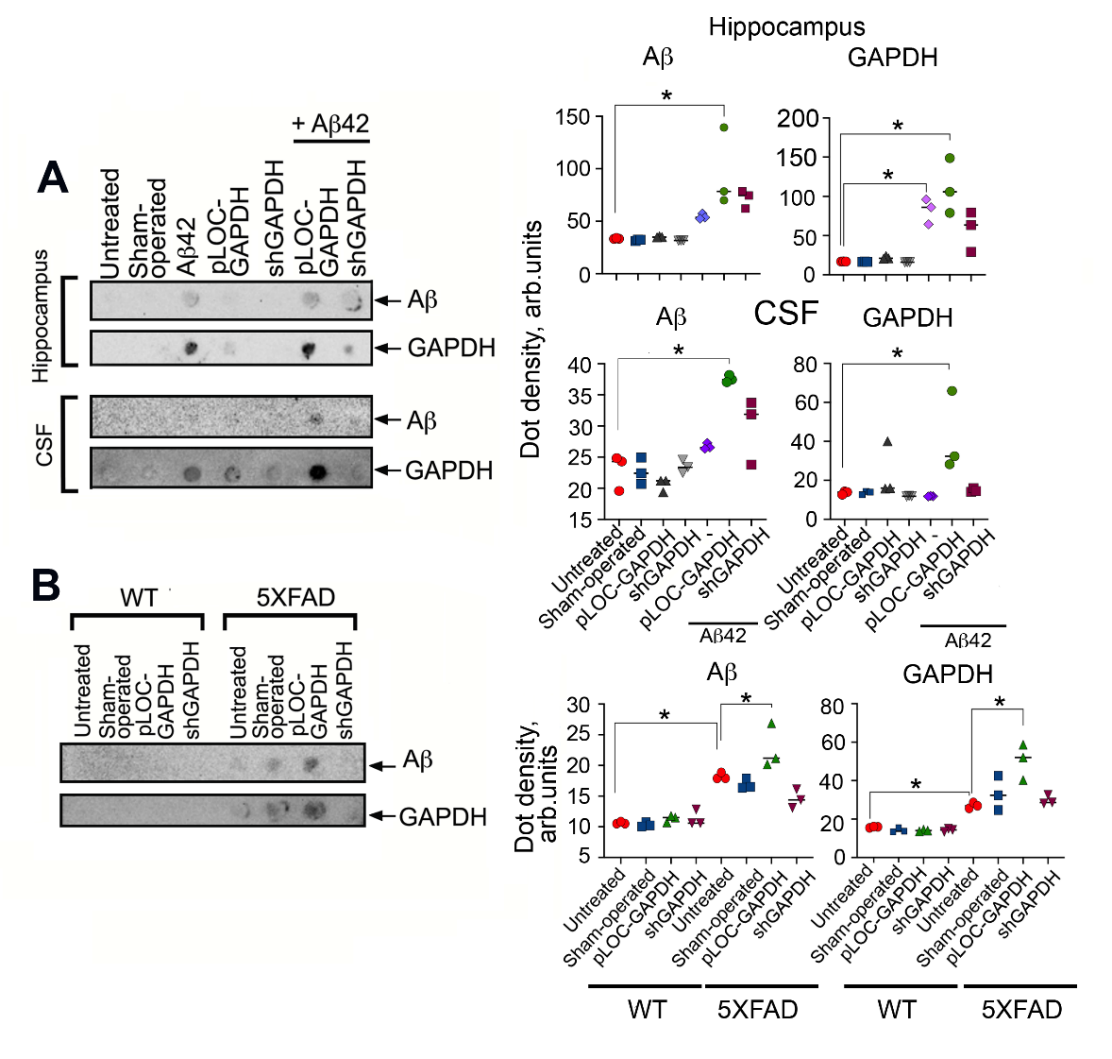
GAPDH expression increases Aβ aggregates in hippocampus and delays Aβ clearance from CSF. (A) Representative filter-trap assays (left panel) of hippocampus tissue and CSF samples of rats from all experimental groups taken two months after injection of lentiviral pLOC-GAPDH or shGAPDH RNA and Aβ42 (*n* = 3 in each group). (B) Representative filter-trap assays of hippocampi from wt and 5XFAD mice, sham-operated or injected with lentiviruses for up- and down-regulation of GAPDH (*n* = 3 in each group). Right panels show dot intensity data from (*A*) and (*B*). *, *p < 0.05*.

We next examined the behavioral effect of endogenous GAPDH in AD model animals. Wistar male rats at P180 with high and low GAPDH were tested in a Morris water maze at P240 to investigate learning function. In both models of AD, animals with elevated levels of GAPDH in the hippocampus demonstrated significant cognitive impairment. The platform searching time for rats injected with pLOC-GAPDH+Aβ42 was, respectively, 1.6-fold and 2.4-fold longer than for the Aβ42 group and the untreated group (Fig. 5A). In the 5XFAD mouse model, animals injected with pLOC-GAPDH demonstrated a 2-fold delay in searching comparing to that in untreated animals. In contrast, animals injected with shGAPDH displayed a shorter searching time than untreated animals (Fig. 5B).

To correlate the degree of memory deficit with GAPDH cytotoxicity, we obtained CSF from each group of rats and estimated its effect on SH-SY5Y neuroblastoma cell survival. As expected, the most toxic CSF was obtained from the animals injected with pLOC-GAPDH+Aβ42, killing 22.2 ± 3.1% of the cells (Fig. 5C).

The enhanced cytotoxicity of CSF obtained from AD patients was linked to aggregation of Aβ-GAPDH (Fig. 1). To confirm this finding in the AD animal models, the amount of Aβ-GAPDH aggregates in CSF and hippocampi from all treated animals was estimated using ultrafiltration. SDS-insoluble aggregates containing both Aβ42 and GAPDH were found in the hippocampus and CSF of rats injected with Aβ42 and pLOC-GAPDH+Aβ42 (Fig.6A). Likewise, the level of Aβ42 and GAPDH aggregation in 5XFAD mice injected with pLOC-GAPDH was significantly higher than in control mice (Fig. 6B).

### GAPDH ligand reduces Aβ aggregation and ameliorates spatial memory in 5XFAD mice

**Figure 7.**
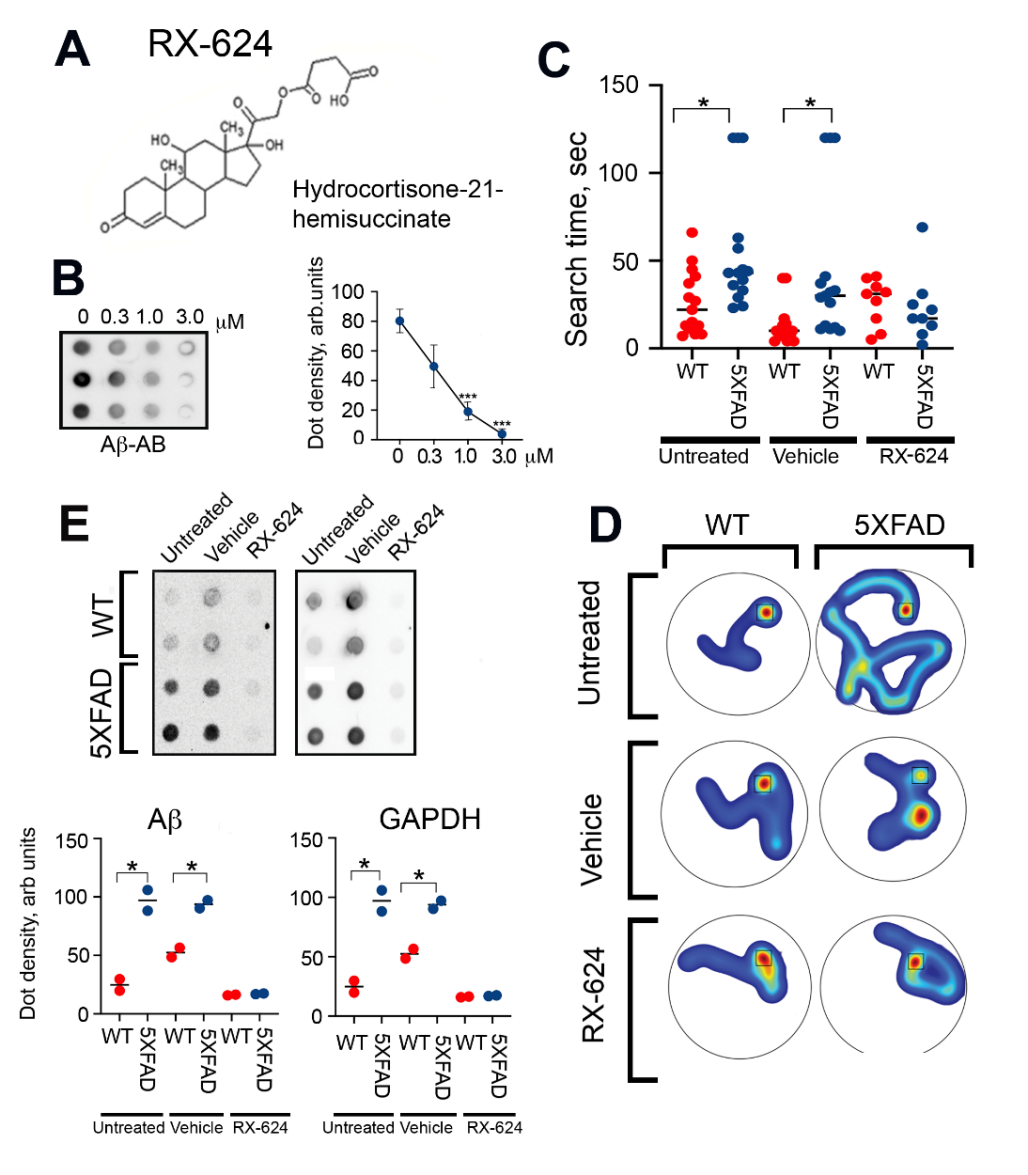
RX-624 improved spatial memory and decreases Aβ-GAPDH aggregates in hippocampus of 5XFAD mice. (A) Chemical structure of RX-624. (B) Ultrafiltration assay with a mixture of Aβ42, GAPDH, and tTG in the presence of the indicated concentrations of RX-624. Representative filter (left panel) was probed with antibody against Aβ. Dot intensity data from three independent experiments (right panel), *p<0,01. (C) Water-maze memory test. WT and 5XFAD mice were each divided into 3 groups of 10 animals. The first group did not receive any treatment, the second was treated with 100 µl of vehicle, and the third with 100 µl of RX-624 (2 mg/kg/injection). Times needed to find a submerged platform was measured; * p < 0.05. (D) The swimming trajectories of animals were performed with EthoVision XT14.0 software. Location of invisible platform indicated by squares. (E) Aβ-GAPDH aggregation test. The hippocampi of two mice from each experimental group were isolated and subjected to ultrafiltration. The representative membranes were incubated with antibodies against Aβ and GAPDH (upper panel). Quantitation was done using TotalLab Quant software (lower panels); * p < 0.05.

Previously, we have showed that a cortisol derivative (Fig. 7A), hydrocortisone 21-hemisuccinate (RX-624), specifically binds GAPDH and reduces its aggregation [36]. To test whether RX-624 is capable of reducing aggregation of Aβ-GAPDH complexes as well, we mixed Aβ42, GAPDH and tTG in the presence of 0.3, 1.0 and 3.0 µM of RX-624 followed by ultrafiltration and immunoblotting. RX-624 diminished aggregation of Aβ-GAPDH complexes in a dose-dependent manner, reaching 80-fold reduction at 3.0 µM (Fig. 7B).

To test the effectiveness of RX-624 in animal models of AD, wt and 5XFAD mice were divided into three groups: no treatment, vehicle, and RX-624 (2 mg/kg/injection). The treatment started at P70 of age and lasted for next 150 days, one injection per week. Two weeks after the last injection spatial memory of experimental mice was evaluated using the Morris water maze. 5XFAD mice treated with RX-624 demonstrated significant memory recovery; the platform searching time was similar to that of wt mice (22.7 ± 4.4 vs. 26.2 ± 6.6 sec) and was greatly reduced compared to that of untreated or vehicle-treated 5XFAD mice (Fig. 7C). Analysis of swimming paths performed following the instructions given by Gehring et al. [37], also demonstrated that the learning capacity of 5XFAD mice treated with RX-624 approached that of wt mice (Fig. 7D).

Next, the hippocampi from two animals of all three groups were isolated and subjected to ultrafiltration. SDS-insoluble aggregates containing both Aβ and GAPDH were detected in control 5XFAD animals, whereas the hippocampi of mice treated with RX-624 were completely free of aggregates, demonstrating the crucial role of GAPDH in promoting the formation of extracellular amyloid complexes (Fig.7E).

## DISCUSSION

Prion-like transmission of Aβ oligomers and tau fibrils within the brain is thought to account, at least in part, for cell death in AD [38,39]. The late stage of AD pathogenesis is characterized by massive neuronal cell death and persistent accumulation of extracellular Aβ in the brain, apparently due to impaired clearance [40]. Recently, the presence of exogenous soluble aggregates of Aβ in CSF of AD patients has been associated with enhanced neurotoxicity [41]. We argue that in addition to Aβ, CSF contains proteins leaking from dying brain cells. Together they form soluble and insoluble aggregates typical of many neurodegenerative pathologies. In this study we focused on GAPDH, an enzyme known for its propensity to form aggregates when oxidized or complexed with mutant proteins [42].

We found that the amount of soluble GAPDH and insoluble GAPDH complexes with Aβ in the CSF from patients with various stages of AD directly correlated with the severity of the disease; the increase was apparent at moderate and severe stages of AD, as classified by MMSE. Remarkably, the cytotoxic activity of CSF samples increased concurrently with the pathology index, suggesting that extracellular GAPDH promotes AD pathogenesis. Indeed, the GAPDH depletion reduced CSF cytotoxicity, particularly in the samples from ‘Severe’ group of AD patients. We hypothesized that the cytotoxic effect of GAPDH aggregation or co-aggregation with Aβ occurs when the enzyme concentration in extracellular space, CSF, or interstitial fluid reaches a threshold level that results in aggregation. FRET analysis directly supports the existence of stable complexes between Aβ and GAPDH. We detect them as early as the ‘Mild AD’ stage, but find much more of them at ‘Moderate’ and ‘Severe’ AD stages. Thus, the Aβ-GAPDH complexes may serve as a reliable biomarker of AD progression, comparable to other already established markers like CSF amyloid beta 42 (Aβ42), total tau (t-tau), and tau phosphorylated at threonine 181 (p-tau [43], or recently proposed ones like neurogranin/β-site APP-cleaving enzyme 1 (Ng/BACE1) [44].

It has been proposed that the non-ordered assembly of GAPDH aggregates and Aβ oligomers cause depolarization of mitochondrial membranes, ultimately leading to apoptosis of neurons in transgenic mice [45, 46]. In contrast, we present evidence for covalently bonded extracellular Aβ-GAPDH complexes. Our results indicate that such complexes constitute a major fraction of Aβ-GAPDH aggregating material. The formation of these complexes requires tTG activity; inhibition of tTG with cystamine prevents stable co-aggregation. Moreover, we show that tTG stabilizes Aβ-GAPDH complexes via specific covalent bonding between Aβ Q15 and GAPDH lysines. We further show that these covalently bound complexes largely account for Aβ toxicity in animal models of AD.

Overexpression of GAPDH impaired cognitive function in 5XFAD mice. As expected, apoptosis played a significant role in neuronal death in these animals. The extent of apoptosis was comparable to that observed in animals’ cells treated with excessive Aβ to trigger an oxidative apoptotic cascade [47, 48]. Consistently, the cytotoxicity of CSF from animals that overproduce GAPDH was substantially higher than that of control animals. Recently, GAPDH was shown to be released from PC-12HttQ103 rat neuroblastoma cells in complex with long poly-Q chains [16]. Analogous to the increased cytotoxicity of extracellular GAPDH-poly-Q complexes, we propose that a similar phenomenon occurs in AD models. It appears that irrespective of the initial cause of cellular death, the released GAPDH augments the cytotoxicity of pathogenic misfolded polypeptides.

The increased accumulation of Aβ aggregates in hippocampi of animals that overexpress GAPDH is likely due to compromised Aβ clearance. Aβ clearance is considered to be a critical part of AD etiology [40]. Aβ itself has a relatively short half-life in the brain; approximately 8 hours in human CSF [49]. Proteins like ApoA-I, transthyretin, complement protein C3 [50], or acetylcholinesterase [51] have been shown to promote the retention of Aβ in the extra-neuronal space. GAPDH appears to act as a major AD potentiator via a conceptually similar pathway.

Emerging as a significant pathological factor in the late stages of AD, GAPDH may also be used as a promising therapeutic target. Indeed, we show here that hydrocortisone 21-hemisuccinate (RX-624), which specifically binds GAPDH and diminishes its aggregation [36], has drastically reduced the accumulation of aggregated Aβ in hippocampi of 5XFAD mice and greatly improved their spatial memory. Earlier, our group has reported that RX-624 also reduces the cytotoxicity of the extracellular GAPDH-polyQ complexes [36]. Thus RX-624 may be a constituent of a novel class of anti-neurodegeneration therapeutic molecules that diminish the propagation of cytotoxic heterologous protein aggregates, a hypothesis that warrants further exploration in other animal models involving larger mammals.

In summary, we demonstrate here that upon neuronal cell death the most ubiquitous housekeeping protein, GAPDH, acts as a promoter of AD pathogenesis by forming a covalent complex with extracellular Aβ. The amount of Aβ-GAPDH in CSF correlates well with the AD progression and is, therefore, a potentially useful marker for AD diagnostics. Despite setbacks in the clinical treatment of late-stage AD, monoclonal antibodies that neutralize the aggregation-prone and toxic motifs of Aβ remain the most promising experimental anti-AD drugs [52]. Our data also suggest that GAPDH inhibitors/binders, such as RX-624, may render those drugs more efficient in the therapy of AD-associated pathologies.

## Supplementary Materials

The Supplemenantry data can be found online at: www.aginganddisease.org/EN/10.14336/AD.2020.1230.


