# Supplementary material for: Extracellular GAPDH Promotes Alzheimer Disease Progression by Enhancing Amyloid-β Aggregation and Cytotoxicity
Source: Aging Dis. 2021 Aug 1;12(5):1223–37. doi: 10.14336/AD.2020.1230 (PMC8279520; doi:10.14336/AD.2020.1230)
Supplement: Supplementary file 1 [file AD-12-5-1223-s.pdf]

SUPPLEMENTARY DATA

Extracellular GAPDH Promotes Alzheimer Disease Progression by Enhancing Amyloid-β Aggregation and Cytotoxicity

Vladimir F. Lazarev[1,2], Magda Tsolaki[3], Elena R. Mikhaylova[1], Konstantin A. Benken[4], Maxim A. Shevtsov[1,5], Alina D. Nikotina[1], Mirna Lechpammer[6], Vladimir A. Mitkevich[2], Alexander A. Makarov[2], Alexey A. Moskalev[2,7], Sergey A. Kozin[2], Boris A. Margulis[1*], Irina V. Guzhova[1*], Evgeny Nudler[6,8*]

© 2020. Lazarev VF et al. Published online at http://www.aginganddisease.org/EN/10.14336/AD.2020.1230

SUPPLEMENTARY DATA

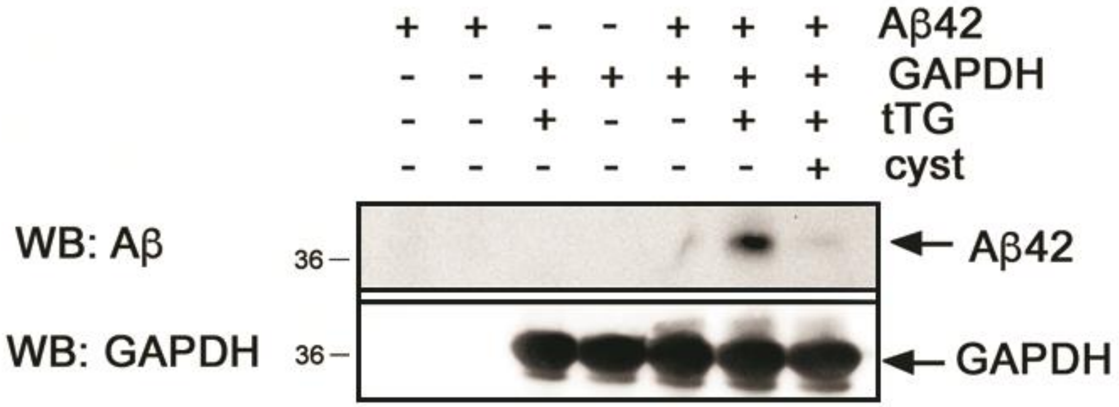

Supplementary Figure 1. GAPDH and Aβ42 form SDS insoluble complex in the presence of tTG.

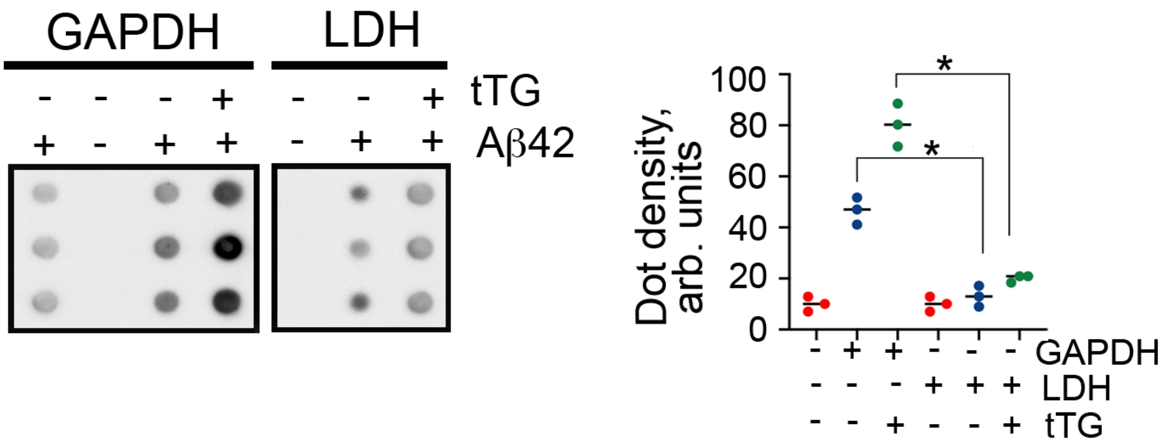

Supplementary Figure 2. An arbitrary cytosolic protein (LDH) released from dying cells does not form insoluble aggregates with Aβ.

© 2020. Lazarev VF et al. Published online at http://www.aginganddisease.org/EN/10.14336/AD.2020.1230

SUPPLEMENTARY DATA

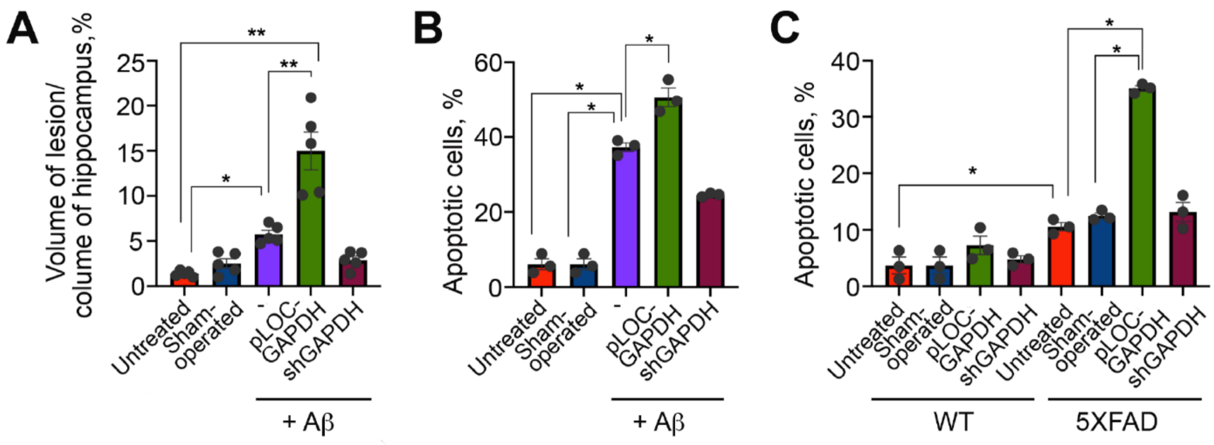

Supplementary Figure 3. Variation of GAPDH content in hippocampus leads to changes in death and apoptosis levels in rat and mouse models of AD. **(A)** Lesions volume of rats two months after injection of Aβ42 together with lentiviral pLOC-GAPDH or Aβ42 together with lentiviral shGAPDH RNA, presented on Fig.4C measured with the aid of TotalLab software (n = 3 for each group); *, $p < 0.05$; **, $p < 0.01$. **(B)** The prevalence of apoptotic cells in rat hippocampi was calculated as the ratio of TUNEL-positive cells to DAPI-stained cells on histological slices using TotalLab software (n = 3 for each group), see Fig. 4D. **(C)** The prevalence of apoptotic cells in mouse hippocampi presented on Fig. 4E was calculated as in (B) (n = 3 for each group); *, $p < 0.05$.

Supplementary Table 1. Cohort of MCI and AD patients used for GAPDH detection in CSF.

#	Stage of Disease	Number of patients in group	Average age	MMSE, range
**1**	MCI	n=22; 12 males, 10 females	69,4±2,5	26-30
**2**	Mild AD	n=41; 22 males, 19 females	75,5±1,1	21-25
**3**	Moderate AD	n=49; 26 males, 23 females	75,6±0,9	12-20
**4**	Severe AD	n=51; 31 males; 20 females	74,8±3,5	0-11

Supplementary Table 2. Cohort of MCI and AD disease patients used for detecting Aβ-GAPDH aggregates in CSF.

#	Stage of Disease	Number of patients in group	Average age	MMSE, range
**1**	MCI	n=6; 4 males, 2 females	71,5±2,5	26-28
**2**	Mild AD	n=8; 6 males, 2 females	76±2,2	21-25
**3**	Moderate AD	n=7; 3 males, 4 females	78,3±2,4	12-20
**4**	Severe AD	n=6; 3males; 3 females	74,8±3,5	0-11

© 2020. Lazarev VF et al. Published online at http://www.aginganddisease.org/EN/10.14336/AD.2020.1230
